# Health-Information Technology Use in People with Multimorbidity: National Health Interview Survey 2022-2023

**DOI:** 10.1093/geroni/igaf122.3216

**Published:** 2025-12-31

**Authors:** Binu Koirala, Arum Lim, Chitchanok Benjasirisan, Soo Hyun Kim, Asma Rayani, Cheryl Himmelfarb, Patricia Davidson

**Affiliations:** Johns Hopkins University, Baltimore, Maryland, United States; Johns Hopkins University School of Nursing, Baltimore, Maryland, United States; Johns Hopkins University, Baltimore, Maryland, United States; Johns Hopkins University, Baltimore, Maryland, United States; Johns Hopkins University, Baltimore, Maryland, United States; Johns Hopkins University, Baltimore, Maryland, United States; University of New South Wales Sydney, Kensington, New South Wales, Australia

## Abstract

Multimorbidity is a key healthcare challenge in the US and highly prevalent among older adults. Health-information technology (HIT) use has been identified as a valuable tool to facilitate multimorbidity management. This study aims to explore the characteristics associated with HIT use among individuals with multimorbidity. A cross-sectional analysis of 2022-2023 National Health Interview Survey (n = 41,194) data was conducted. Presence of multimorbidity was defined as the self-reported history of ≥ 2 chronic conditions: diabetes, hypertension, asthma, stroke, cancer, chronic obstructive pulmonary disease, heart disease (coronary heart disease, heart attack), weak/failing kidneys, and chronic liver conditions. HIT use was defined as any of three aspects of internet use for: health information, communicating with the doctor’s office, and test results. Survey-weighted Poisson regression models were developed for statistical analysis. Majority of individuals with multimorbidity [mean (SD) age: 59(0.19) years] reported often use of the internet for all three aspects of HIT use. In an adjusted analysis, characteristics associated with a lower prevalence ratio of HIT use were older age; male; not married; of a racial and ethnic minority group; lower education and income; no insurance; no usual place for medical care; and reporting excellent perceived health status. Compared to adults aged 18-64 years, adults ≥65 years group were 23% less likely to report HIT use (APR: 0.77, 95% CI: 0.74-0.79). Although HIT use is common among adults with multimorbidity, social determinants of health and age affect usage. Careful consideration of these factors is essential when designing a digital health intervention, particularly for older adults.

